# Association Between Preoperative US, Elastography Features and Prognostic Factors of Papillary Thyroid Cancer With BRAF^V600E^ Mutation

**DOI:** 10.3389/fendo.2019.00902

**Published:** 2020-01-22

**Authors:** Jun-Mei Xu, Yong-Jun Chen, Yuan-Yuan Dang, Man Chen

**Affiliations:** ^1^Department of Medical Ultrasound, Tongren Hospital, Shanghai Jiao Tong University School of Medicine, Shanghai, China; ^2^Department of Medical Ultrasound, Shanghai Tenth People's Hospital, Tong Ji University, Shanghai, China

**Keywords:** thyroid cancer, prognosis, ultrasound, acoustic radiation force impulse, elastography

## Abstract

**Purpose:** To investigate the value of US and elastography for predicting prognostic factors of papillary thyroid cancer (PTC) in the positive BRAF^V600E^ Mutation group.

**Materials and Methods:** A total of 116 BRAF^V600E^ Mutation patients with PTCs were enrolled in this prospective study, who were preoperatively evaluated by US, US elasticity imaging (EI), and Virtual Touch tissue imaging (VTI) and Virtual Touch tissue quantification (VTQ) of acoustic radiation force impulse (ARFI) imaging. Multivariate logistic regression analysis was performed to assess 23 independent variables for predicting prognostic factors. Diagnostic performance was evaluated with receiver operating characteristic (ROC) curve analysis.

**Results:** Forty-two (36.2%) of 116 PTC patients with BRAF^V600E^ Mutation had central lymph node metastasis (LNM). Nine (7.8%) and fifty-six (48.3%) had lateral LNM and extra-thyroidal extension (ETE), respectively. In multivariate logistic regression analyses, rich internal flow [odds ratio [OR]: 6.66] was the best predictor for central LNM, followed by male sex (OR: 4.22), halo sign absence (OR: 2.78) (all *P* < 0.05). VTQ ratio (OR: 1.57) was the only predictor for lateral LNM (*P* = 0.02). Rich internal flow (OR: 6.33) was the strongest predictor for ETE, followed by male sex (OR: 3.29), halo sign absence (OR: 2.90), and VTQ ratio (OR: 1.63) (all *P* < 0.05).

**Conclusion:** VTQ ratio on ARFI imaging, rich internal flow and halo sign absence on US are the predicting prognostic factors in PTC patients with BRAF^V600E^ Mutation. The specificities were significantly increased by combining ARFI imaging and US features, which has a potential to avoid unnecessary therapeutic neck dissection in the high-risk PTC patients.

## Introduction

Papillary thyroid cancer (PTC) is a common endocrine malignancy, which accounts for 80–85% of all thyroid cancers, and generally displays an indolent course (1, 2). Although the overall 10-years survival rate for patients with PTC is higher than 90%, a minority of patients eventually dies as a result of this disease with regional recurrences and distant metastases (3). Many studies have demonstrated an association of BRAF^V600E^ mutation with aggressive clinic pathologic characteristics and poor prognosis of PTC (4–6). However, several other studies have reported no or a partial association of the BRAF mutation with high-risk pathological characteristics (7, 8). Recently, a report of 455 patients with PTC showed that BRAF^V600E^ mutation was not associated with more advanced TNM stage upon diagnosis (8). The association of the BRAF^V600E^ mutation with more aggressive clinic pathological features in patients with PTC thus remains controversial.

Cervical lymph node metastasis (LNM), extra-thyroidal extension (ETE), and distant metastasis are commonly regarded as the independent risk factors of PTC (9–11). The sensitivity and specificity in detecting cervical LNM by US are superior to those by palpation (12, 13), although the value of diagnosis is moderate (36–72%) in clinical node-negative PTC (14). Recently, some authors reported that absence of thyroid capsule and contact with capsule on US were the independent risk factors for cervical LNM or ETE, but the related features were not evaluated in high-risk PTC patients (15–17). Based on the association between tumor stiffness and clinic pathologic characteristics, US elastic imaging (EI) was also found to be a useful technology for predicting ETE and cervical LNM (18, 19).

As a new US-based elastic technique, acoustic radiation force impulse (ARFI) is emitted from the US transducer instead of using manual compression. ARFI allows qualitative (virtual touch tissue imaging, VTI) and quantitative (virtual touch tissue quantification, VTQ) estimation of the tissue stiffness (20–22). VTQ can be obtained by measuring the time to peak displacement in transverse direction at each lateral location and the shear wave velocity (SWV) within the tissue can be calculated whereas VTI is created by the repetition of the process of tissue displacement along multiple image lines (20–22). Consequently, operator-independence and reproducible of ARFI are more superior to those of conventional EI (16, 23, 24). Although ARFI imaging is useful in predicting cervical LNM (15, 16), there are no studies on the association between ARFI imaging findings and prognosis in the positive BRAF^V600E^ Mutation group. Herein we hypothesized that ARFI imaging might be useful in predicting poor prognosis in the high-risk PTC group. The purpose of our study was to evaluate the value of US and elastography to predict prognostic factors of PTC in the positive BRAF^V600E^ Mutation group.

## Materials and Methods

### Study Design and Patients Involved

**Figure 1:**
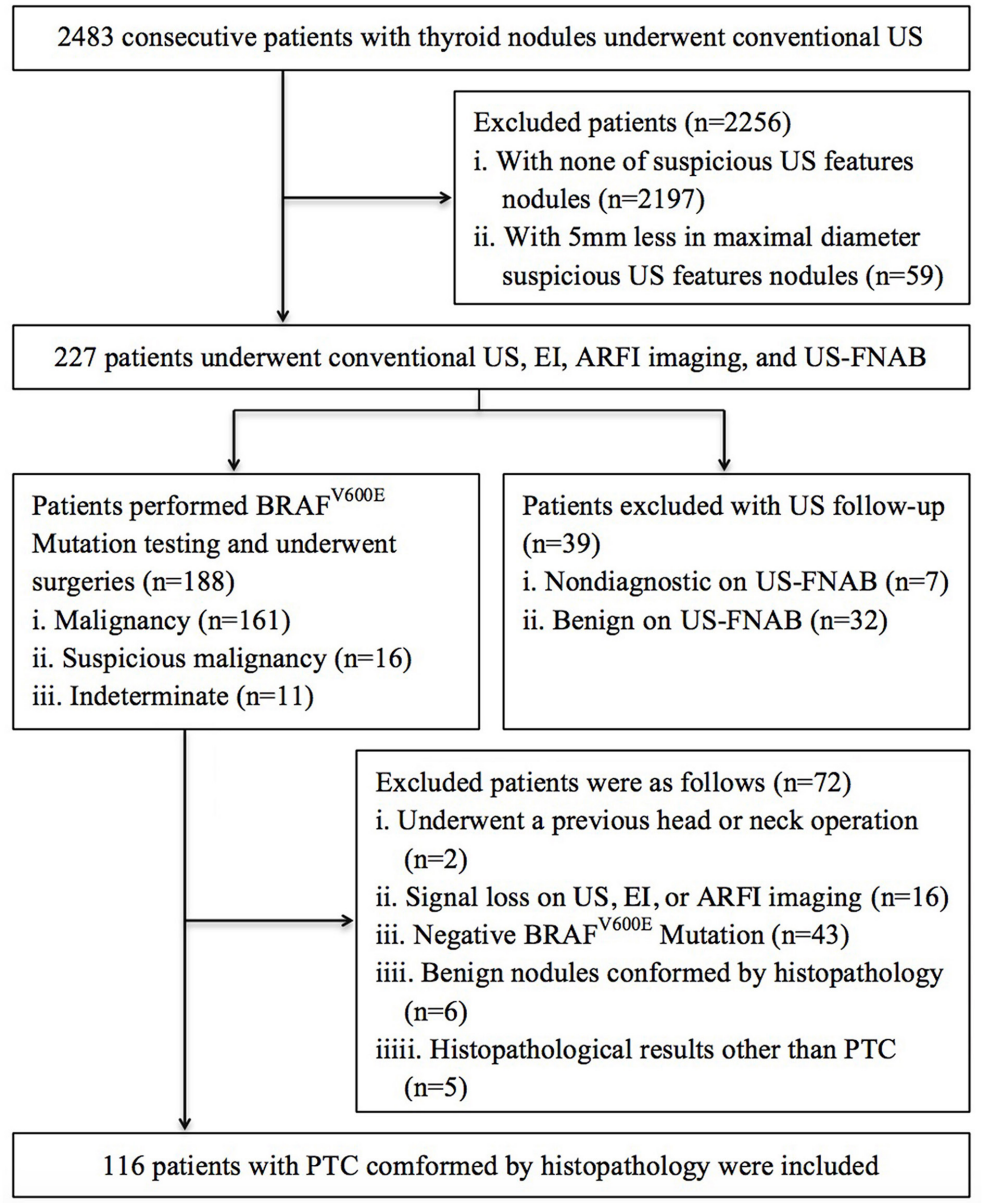
Flowchart of the selection of the patients with PTC.

The ethics committee of university hospital approved this prospective study and informed consent was obtained from all patients for all experimental protocol before their examination. The methods in this study were carried out in accordance with approved guidelines. From October 2015 to September 2016, 2,483 consecutive patients with thyroid nodules underwent US in our institution. Among them, 227 patients underwent EI, ARFI imaging, and fine-needle aspiration cytology (FNAC), if their thyroid nodules had one of suspicious malignancy US features and at least 5 mm in maximal diameter. In patients with multiple suspicious nodules on US, we selected the largest one for analysis. The US features of suspicious malignancy for thyroid nodules were defined as hypo- or marked hypo-echogenicity, irregular or micro-lobulated margins, micro-calcifications, taller than wide shape, halo sign absent, and solid at our institution (12, 20–22). Based on the Bethesda system of cytological diagnosis, the preoperative FNAC outcomes were: (1) 161 nodules were malignant; (2) 16 nodules were suspicious for malignancy; (3) 11 nodules were indeterminate; (4) 7 nodules were no diagnostic; (5) 32 nodules were benign. Thirty-nine patients with 39 no diagnostic and benign nodules were excluded because they selected US follow-up instead of repeated FNAC or surgery. For 188 patients with malignancy/suspicious malignancy and indeterminate nodules, BRAF^V600E^ Mutation testing was performed using the preoperative FNAC specimen, and surgeries were undergone. Excluded patients were as follows: (1) 2 had undergone a previous head or neck operation; (2) 16 had signal loss on US, EI, or ARFI imaging; (3) 43 patients had negative BRAF^V600E^ Mutation; (4) 6 had benign nodules confirmed by histopathology; (5) 5 had histopathological results other than PTC. Finally, 116 patients (32 males and 84 females; mean age 50.4 ± 14.0 years, range 23–77 years) with PTC were included in this study (Figure 1).

### Conventional US, EI, and ARFI Imaging

Conventional US, EI, and ARFI imaging were performed by one radiologist, who had more than 10 years' thyroid imaging experience. All imaging was obtained by using the same S2000 US machine (Siemens Medical Systems, Mountain View, CA) with a 4–9 MHz linear array transducer. The longitudinal and transverse sections were routinely scanned on US, and the maximum diameter of tumor was measured. Findings on conventional B-mode and elastic imaging were prospectively recorded in the radiologic reports. The features of target tumor on US were evaluated as following: size (the largest diameter), location (defined as left-, right-lobe, and isthmus; upper-, mid-, and low-third; Medial-, lateral-, ventral-, dorsal-, and middle-part), internal component (defined as completely solid, cystic portion <25%, and cystic portion ≥25%), echogenicity (defined as marked hypo-, hypo-, iso-/hyper-, mixed-echogenicity comparing with surrounding thyroid parenchyma and nearby strap muscle), internal calcifications (defined as micro-calcifications <2 mm in diameter, macro-calcifications ≥2 mm in diameter, no calcification; when micro-calcifications were in coexistence with the macro-calcifications, which were defined as micro-calcifications), internal flow (rich internal flow, more than three linear on color Doppler US; rare internal flow, dotted signals on color Doppler US; no internal flow, without signals on color Doppler US), shape (regular or irregular), margin (well or poorly defined, with “poorly defined” defined as microlobuated or spiculated margin), halo sign (presence or absence), taller than wide shape or not, capsule contact (presence or absence), capsule involvement (presence or absence).

After the US examination, EI was performed at the target tumor with a light pressure. The quality indicator scale displayed more than 60 to ensure sufficient quality image. Thyroid tumors were evaluated based on a 4-score system according to the description by Asteria and Rubaltelli (25, 26). Score 1 and 2 expressed tumors with high elasticity (i.e., softer component), which indicated that the tumor was benign; score 3 and 4 expressed tumors with low elasticity (i.e., stiffer component), which indicated that the tumor was malignant.

ARFI imaging was subsequently performed using the same machine and probe. Tissue within the region of interest (ROI) is mechanically excited by obtaining short-duration acoustic pulses to generate transverse shear waves. Then VTQ measurement was performed. ROI was placed at the most internal portion of the tumor as possible as to avoid calcified or cystic portions. The tumor and peripheral thyroid tissue were, respectively, measured 7 times in the same location, and the results of shear wave velocity (SWV) were expressed in m/s. The highest and lowest values had been excluded before the average of the remaining 5 values was calculated for the analysis. The stiffness of tissue was more likely to be hard, when the value of SWV increased. In addition, VTQ ratio was calculated, and defined as the average value of tumor tissue divided by the average value of peripheral thyroid tissue. In VTI mode, the gray scale images and VTI images were displayed simultaneously in a split-screen mode. Two gray scale values (black or white) displayed in VTI image. The white scale indicated that areas were greatest strain (i.e., softest component); correspondingly, the black scale indicated that areas were less or no strain (i.e., hardest component). According to the Xu's VTI grading method, VTI images were divided into 6 grades (15, 16, 20, 21). The stiffness of tissue was more likely to be hard, when the grade of VTI images increased. In addition, the VTI area ratio (VAR) of the tumors was calculated and defined as the area of the tumor on VTI image divided by the area on US image (15, 16, 22).

All the US, EI, and ARFI images were independently analyzed by two skilled radiologists who both had 12 years' experience in thyroid US, 8 years' experience in thyroid EI and 5 years' experience in thyroid ARFI. The clinic and pathology of the patients were in a blind manner. In cases with disagreement, the final decision was assigned with a third senior investigator who had 25 years' experience in thyroid US, 10 years' experience in thyroid EI and 6 years' experience in thyroid ARFI.

### DNA Extraction and Detection of the BRAF Mutation in FNAC Specimens

FNAC was performed by the radiologist who carried out US, EI and ARFI imaging examination. The target of FNAC was the most suspicious US feature for malignancy in the tumor. Free hand FNAC was performed at least three times for each tumor by using a 23-gauge needle attach to 2-mL disposable plastic syringe. Samples obtained from the first passage were smeared and placed immediately in 95% alcohol for Papanicolaou staining. The remaining material was rinsed in saline for cellblock processing. Cytopathologist was not on site during biopsies. Real-time PCR and direct sequencing were performed for BRAF mutation analysis by genomic DNA extracted from FNAC sample, which were described in previously studies (4, 7).

### Surgery and Reference Standard

The extent of thyroid surgery was determined in accordance with the guidelines of the American Thyroid Association (ATA) (12). Total or near total thyroidectomy was undergone, When patient had multiple, or bilateral tumors, or extrathyroidal invasion. Central compartmental neck dissection including paratracheal, peritracheal, and prelaryngeal lymph nodes (LNs) was performed routinely, when thyroid malignancy was determined by preoperative cytology or intraoperative frozen-section analysis. Based on the clinical diagnosis with cervical LNM or not, lateral compartmental dissection, including levels 2, 3, 4, and anterior 5, was selectively performed. If suspicious LN was found during surgery, LN sampling and frozen-section examinations were performed. Then lateral compartmental dissection was performed after metastasis was confirmed.

The final reference was obtained according to the histopathological results. Histological subtypes and tumor node metastasis (TNM) staging were evaluated according to the American Joint Committee on Cancer/International Union against Cancer pathologic TNM classification criteria (27). Two or more malignant foci in one lobe were defined as Multifocality. One or more malignant foci in both lobes were defined as bilaterality. ETE was defined as extrathyroid infiltration of the fat tissue and/or of the muscle surrounding or cervical LNM (including central LNM and lateral LNM).

### Data and Statistical Analysis

According to the presence or absence of central LNM or lateral LNM or ETE, patients were divided into three groups. Chi-square test or Fisher's exact test was used to analyze Categorical variables. Independent two-sample *t* test was used to analyze the difference of continuous variables. If continuous variables were in normal distribution, mean ± standard deviation (SD) was expressed, otherwise as range. Stepwise Multivariate logistic regression was performed to determine the risk factors associated with central LNM/lateral LNM/ ETE. Confidence intervals (CIs) for proportions of Odds ratios (ORs) were expressed as two-sided exact binomial 95% CIs. Receiver-operating characteristic (ROC) curves were constructed to evaluate the significant factors on multivariate logistic regression analysis. Based on the corresponding area under the ROC curve (Az), the sensitivity and specificity of each significant independent or combined variable in prediction of central LNM or lateral LNM or ETE were calculated. Two-sided *P* values of <0.05 were defined as statistically significant difference. All statistical tests were performed with SPSS software (version 19.0; SPSS Inc., Chicago, IL).

## Results

### Histopathologic Findings

Based on the histopathological examination, 116 patients with PTC were confirmed. Among them, 35 patients (30.2%) had multifocal foci and 24 (20.7%) had bilateral lesions. ETE was found in 56 (48.3%) of the patients, among whom 42 (36.2%) had central LNM, 4 (3.4%) had lateral LNM, 5 (4.3%) had both central and lateral LNM, 4 (3.4%) had thyroid capsule involvement, 1 (0.9%) had both capsule and striated muscle involvement. The mean size of the index malignancy on histopathology was 11.6 ± 6.1 mm (median, 9.0 mm; range, 5–36 mm). Distant metastasis was not found in any of the patients.

### Patient's Basic Characteristics

The basic characteristics of patients were listed in Table 1. Patient gender was significantly different between the patients with and without central LNM or ETE (*P* < 0.05), which did not show significant difference between the patients with and without lateral LNM (*P* = 0.445). Patient age, nodule size, nodule location, multiple nodules, multiple cancers, bilateral cancers, and chronic lymphocytic thyroiditis did not achieve significant difference between with and without central LNM or lateral LNM or ETE (all *P* > 0.05).

**Table 1:** Basic characteristics of the patients with papillary thyroid cancer.

**Characteristics**	**Central LNM (*****N*** **=** **116)**		***P***	**Lateral LNM (*****N*** **=** **116)**		***P***	**ETE (*****N*** **=** **116)**		***P***
	**Yes (*N* = 42)**	**No (*N* = 74)**		**Yes (*N* = 9)**	**No (*N* = 107)**		**Yes (*N* = 56)**	**No (*N* = 60)**	
**Patients**									
Mean age (years)	47 ± 14	52 ± 14	0.058	51 ± 16	50 ± 14	0.918	48 ± 14	52 ± 14	0.131
Range of age (min-max)	23–76	25–77		31–77	23–77		23–77	25–77	
Gender			0.006			0.445^*^			0.021
Male, *N* (%)	18 (42.9)	14 (18.9)		1 (11.1)	31 (29.0)		21 (37.5)	11 (18.3)	
Female, *N* (%)	24 (57.1)	60 (81.1)		8 (88.9)	76 (71.0)		35 (62.5)	49 (81.7)	
Multiple nodules			0.265			0.548^*^			1.000
Yes, *n* (%)	29 (69.0)	58 (78.4)		8 (88.9)	79 (73.8)		42 (75.0)	45 (75.0)	
No, *n* (%)	13 (31.0)	16 (21.6)		1 (11.1)	28 (26.2)		14 (25.0)	15 (25.0)	
Multiple cancers			0.576			0.553^*^			0.395
Yes, *n* (%)	14 (33.3)	21 (28.4)		4 (44.4)	31 (29.0)		19 (33.9)	16 (26.7)	
No, *n* (%)	28 (66.7)	53 (71.6)		5 (55.6)	76 (71.0)		37 (66.1)	44 (73.3)	
Bilateral cancers			0.532			0.585^*^			0.268
Yes, *n* (%)	10 (23.8)	14 (18.9)		3 (33.3)	21 (19.6)		14 (25.0)	10 (16.7)	
No, *n* (%)	32 (76.2)	60 (81.1)		6 (66.7)	86 (80.4)		42 (75.0)	50 (83.3)	
Chronic lymphocytic thyroiditis			0.568			0.075^*^			0.373
Yes, *n* (%)	17 (40.5)	34 (45.9)		7 (77.8)	44 (41.1)		27 (48.2)	24 (40.0)	
No, *n* (%)	25 (59.5)	40 (54.1)		2 (22.2)	63 (58.9)		29 (51.8)	36 (60.0)	
**Nodule**									
Mean size (mm, range)	12.9 ± 7.0	11.0 ± 5.4	0.133	11.4 ± 5.4	11.7 ± 6.2	0.922	12.8 ± 6.8	10.6 ± 5.3	0.052
Range of size (min–max)	6–36	5–28		6–23	5–36		6–36	5–28	
Location			0.199			0.390^#^			0.533
Left, *n* (%)	18 (42.9)	39 (52.7)		5 (55.6)	52 (48.6)		27 (48.2)	30 (50.0)	
Right, *n* (%)	21 (50.0)	34 (45.9)		4 (44.4)	51 (47.7)		26 (46.4)	29 (48.3)	
Isthmus, *n* (%)	3 (7.1)	1 (1.4)		0 (0.0)	4 (3.7)		3 (5.4)	1 (1.7)	
			0.713			0.390			0.560
Upper-third	16 (38.1)	26 (35.1)		5 (55.6)	37 (36.4)		22 (39.3)	20 (33.3)	
Mix-third	9 (21.4)	21 (28.4)		1 (11.1)	29 (27.1)		12 (21.4)	18 (30.0)	
Low-third	17 (40.5)	27 (36.5)		3 (33.3)	41 (36.5)		22 (39.3)	22 (36.7)	
			0.860			0.529^#^			0.845
Middle	5 (11.9)	8 (10.8)		0 (0.0)	13 (12.1)		6 (10.7)	7 (11.7)	
Medial	6 (14.3)	12 (16.2)		2 (22.2)	16 (15.0)		8 (14.3)	10 (16.7)	
Lateral	9 (21.4)	14 (18.9)		3 (33.3)	20 (18.7)		13 (23.2)	10 (16.7)	
Ventral	13 (31.0)	18 (24.3)		1 (11.1)	30 (28.0)		16 (28.6)	15 (25.0)	
Dorsal	9 (21.4)	22 (29.7)		3 (33.3)	28 (26.2)		13 (23.2)	18 (30.0)	

N, the number of patients; LNM, lymph node metastasis; ETE, extrathyroidal extension;

* Continuity Correction;

# *Fisher's Exact Test*.

### Conventional US, EI, and ARFI Elastography Features

**Figure 2:**
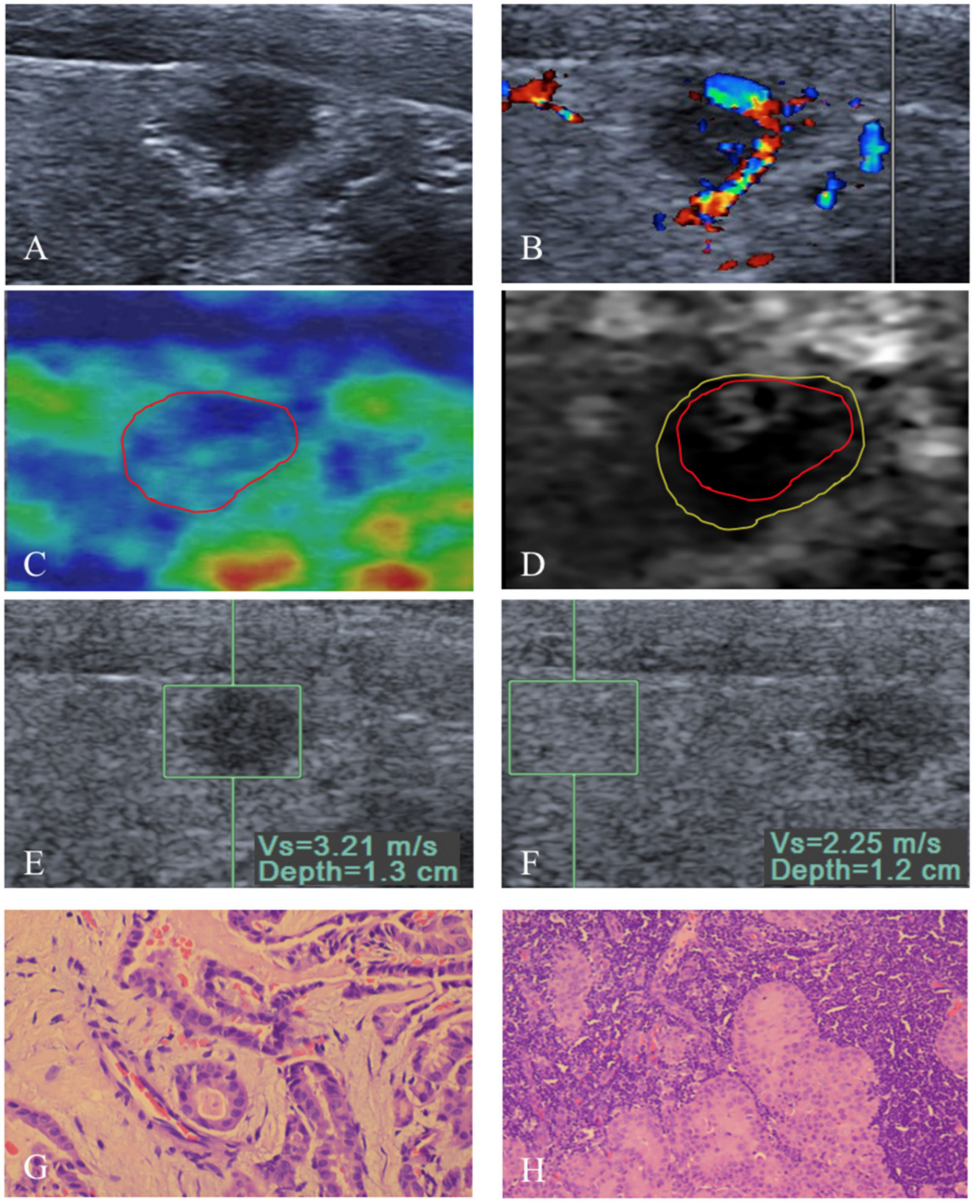
An 8 mm papillary thyroid carcinoma in a 53-year-old man. **(A)** US image show that the tumor is in contact with the adjacent thyroid capsule, and there is halo sign absence. **(B)** Rich intranodular and peripheral flow is found on color Doppler flow imaging of the tumor. At elastography, **(C)** EI score is defined as 2 (red solid line). **(D)** VTI grade is defined as IV (yellow solid line), and the VTI area radio (line _yellow_/line _red_) is 1.32. **(E,F)** SWV of 3.21 m/s is displayed on VTQ, and the VTQ ratio (SWV_E_/SWV_F_) is 1.43. **(G)** PTC was confirmed by pathology (hematoxylin–eosin stain, ×400 magnification). **(H)** Central lymph node metastasis (hematoxylin–eosin stain, ×200 magnification) was confirmed by pathology after total thyroidectomy with cervical lymph node dissection.

**Figure 3:**
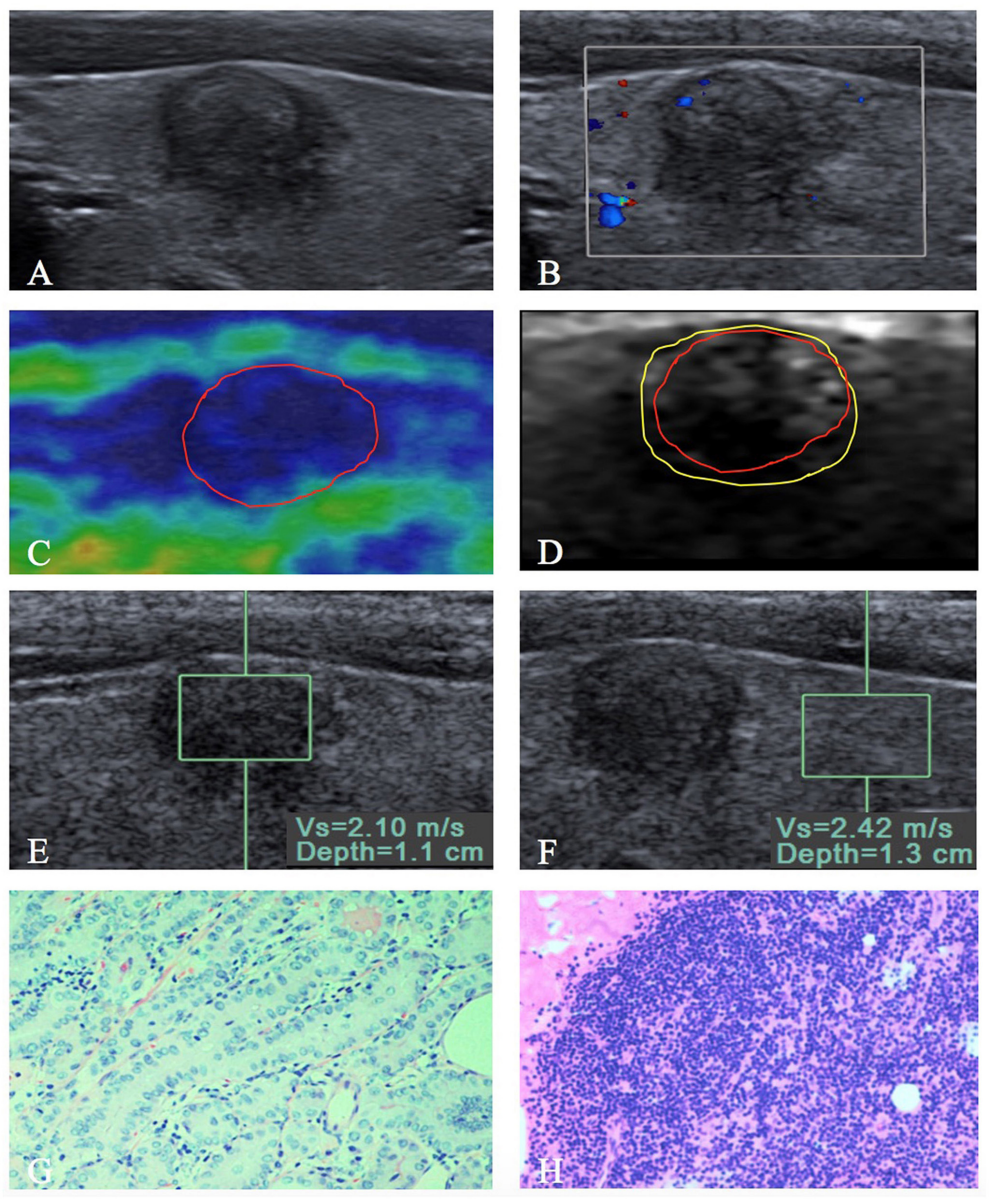
A 9 mm papillary thyroid carcinoma in a 35-year-old woman. **(A)** US image show that the tumor is in contact with the adjacent thyroid capsule, and there is halo sign present. **(B)** Rare intranodular flow is found on color Doppler flow imaging of the tumor. At elastography, **(C)** EI score is defined as 4 (red solid line). **(D)** VTI grade is defined as IV (yellow solid line), and the VTI area radio (line _yellow_/line _red_) is 1.21. **(E,F)** SWV of 2.10 m/s is displayed on VTQ, and the VTQ ratio (SWV_E_/SWV_F_) is 0.87. **(G)** PTC was confirmed by pathology (hematoxylin–eosin stain, ×200 magnification). **(H)** PTC without extrathyroidal extension (hematoxylin–eosin stain, ×200 magnification) was confirmed by pathology after total thyroidectomy with central lymph node dissection.

With regard to the conventional US features, capsule involvement was significantly associated with ETE (*P* < 0.05), but there was not associated with central and lateral LNM (both *P* > 0.05). Rich internal flow was both significantly associated with central LNM and ETE (*P* < 0.05), whereas there was not significantly associated with lateral LNM (*P* > 0.05). Solid component, marked hypo-echogenicity, micro-calcification, irregular shape, poorly defined margin, halo sign absence, taller than wide shape, and capsule contact were all not significant in patients with central LNM or lateral LNM or ETE (all *P* > 0.05) (Table 2, Figures 2, 3).

**Table 2:** The US features of the papillary thyroid cancer.

**Features**	**Central LNM (*****N*** **=** **116)**		***P***	**Lateral LNM (*****N*** **=** **116)**		***P***	**ETE (*****N*** **=** **116)**		***P***
	**Yes (*N* = 42)**	**No (*N* = 74)**		**Yes (*N* = 9)**	**No (*N* = 107)**		**Yes (*N* = 56)**	**No (*N* = 60)**	
Component			0.979			1.000^#^			0.805
Cystic ≥ 25%, *n* (%)	2 (4.8)	3 (4.1)		0 (0.0)	5 (4.7)		2 (3.6)	3 (5.0)	
Cystic < 25%, *n* (%)	1 (2.4)	2 (2.7)		0 (0.0)	3 (2.8)		1 (1.8)	2 (3.3)	
Solid, *n* (%)	39 (92.8)	69 (93.2)		9 (100.0)	99 (92.5)		53 (94.6)	55 (91.7)	
Echogenicity			0.297			0.253^#^			0.449
Marked hypo-, *n* (%)	14 (33.3)	38 (51.4)		3 (33.3)	49 (45.8)		21 (37.5)	31 (51.7)	
Hypo-, *n* (%)	18 (42.9)	22 (29.7)		3 (33.3)	37 (34.6)		21 (37.5)	19 (31.7)	
Iso-/Hyper-, *n* (%)	1 (2.4)	2 (2.7)		0 (0.0)	3 (2.8)		2 (3.6)	1 (1.7)	
Mixed-, *n* (%)	9 (21.4)	12 (16.2)		3 (33.3)	18 (16.8)		12 (21.4)	9 (15.0)	
Calcification			0.704			0.051^#^			0.858
Micro-, *n* (%)	22 (52.4)	44 (59.5)		7 (77.8)	59 (55.2)		33 (58.9)	33 (55.0)	
Macro-, *n* (%)	5 (11.9)	9 (12.1)		2 (22.2)	12 (11.2)		7 (12.5)	7 (11.7)	
No calcification, *n* (%)	15 (35.7)	21 (28.4)		0 (0.0)	36 (33.6)		16 (28.6)	20 (33.3)	
Internal flow			0.015			0.777			0.029
Rich, *n* (%)	19 (45.2)	19 (25.7)		2 (22.2)	36 (33.6)		24 (42.9)	14 (23.3)	
Rare, *n* (%)	17 (40.5)	19 (25.7)		4 (44.4)	42 (39.3)		22 (39.3)	24 (40.0)	
None, *n* (%)	6 (14.3)	26 (35.1)		3 (33.3)	29 (27.1)		10 (17.8)	22 (36.7)	
Irregular shape			0.537			0.772^*^			0.509
Yes, *n* (%)	26 (61.9)	50 (50.0)		5 (55.6)	71 (66.4)		35 (62.5)	41 (68.3)	
No, *n* (%)	16 (38.1)	24 (50.0)		4 (44.4)	36 (33.6)		21 (37.5)	19 (31.7)	
Poorly defined margin			0.313			0.772^*^			0.903
Yes, *n* (%)	30 (71.4)	46 (62.7)		5 (55.6)	71 (66.4)		37 (66.1)	39 (65.0)	
No, *n* (%)	12 (28.6)	28 (37.3)		4 (44.4)	36 (33.6)		19 (33.9)	21 (35.0)	
Halo sign absence			0.192			0.480^*^			0.299
Yes, *n* (%)	29 (69.0)	42 (73.1)		7 (77.8)	64 (59.8)		37 (66.1)	34 (56.7)	
No, *n* (%)	13 (31.0)	32 (26.9)		2 (22.2)	43 (40.2)		19 (33.9)	26 (43.3)	
Taller than wide shape			0.337			0.749^*^			0.084
Yes, *n* (%)	26 (61.9)	39 (56.0)		6 (66.7)	59 (55.1)		36 (64.3)	29 (48.3)	
No, *n* (%)	16 (38.1)	35 (44.0)		3 (33.3)	48 (44.9)		20 (35.7)	31 (51.7)	
Capsule contact			0.742			0.200^#^			0.100
Yes, *n* (%)	34 (81.0)	58 (73.1)		9 (100.0)	83 (77.6)		48 (85.7)	44 (73.3)	
No, *n* (%)	8 (19.0)	16 (26.9)		0 (0.0)	24 (22.4)		8 (14.3)	16 (26.7)	
Capsule involvement			0.309			0.248^*^			0.029
Yes, *n* (%)	12 (28.6)	15 (21.6)		4 (44.4)	23 (21.5)		18 (32.1)	9 (15.0)	
No, *n* (%)	30 (71.4)	59 (78.4)		5 (55.6)	84 (78.5)		38 (67.9)	51 (85.0)	

N, the number of nodules; US, ultrasound; LNM, lymph node metastasis; ETE, extrathyroidal extension;

* Continuity Correction;

# *Fisher's Exact Test*.

The SWV of VTQ was significantly associated with lateral LNM (*P* < 0.05), but there was not associated with central LNM and ETE (both *P* > 0.05). VTQ ratio achieved significant difference in patients with lateral LNM or ETE (both *P* < 0.05), whereas there was no significant difference in patients with central LNM (*P* > 0.05) (Table 3, Figures 2, 3). For VTI and EI, no significant differences presented between PTCs with and without central LNM or lateral LNM or ETE (all *P* > 0.05).

**Table 3:** The EI and ARFI imaging features of the papillary thyroid cancer.

**Features**	**Central LNM (*****N*** **=** **116)**		***P***	**Lateral LNM (*****N*** **=** **116)**		***P***	**ETE (*****N*** **=** **116)**		***P***
	**Yes (*N* = 42)**	**No (*N* = 74)**		**Yes (*N* = 9)**	**No (*N* = 107)**		**Yes (*N* = 56)**	**No (*N* = 60)**	
**ARFI**									
VTI			0.328			0.241^#^			0.411
Grade I, *n* (%)	1 (2.1)	1 (1.5)		0 (0.0)	2 (1.9)		1 (1.8)	1 (1.7)	
Grade II, *n* (%)	1 (2.1)	6 (8.7)		2 (22.2)	5 (4.7)		3 (5.4)	4 (6.7)	
Grade III, *n* (%)	2 (6.4)	5 (5.8)		1 (11.1)	6 (5.6)		3 (5.4)	4 (6.7)	
Grade IV, *n* (%)	16 (40.4)	38 (50.7)		3 (33.3)	51 (47.7)		21 (37.5)	33 (55.0)	
Grade V, *n* (%)	16 (36.2)	19 (26.1)		2 (22.2)	33 (30.8)		21 (37.5)	14 (23.3)	
Grade VI, *n* (%)	6 (12.8)	5 (7.2)		1 (11.1)	10 (9.3)		7 (12.5)	4 (6.7)	
VAR			0.221			0.178			0.057
Mean	1.3 ± 0.4	1.2 ± 0.2		1.4 ± 0.4	1.3 ± 0.3		1.3 ± 0.4	1.2 ± 0.2	
Range (min-max)	0.8–3.4	0.9–2.1		1.0–2.1	0.8–3.4		0.8–3.4	0.9–1.8	
VTQ			0.857			0.040			0.100
Mean SWVs (m/s),	4.1 ± 2.4	4.0 ± 2.2		5.5 ± 2.8	3.9 ± 2.2		4.4 ± 2.5	3.7 ± 2.0	
Range of SWVs, (min–max)	0.8–8.4	0.8–8.4		2.6–8.4	0.8–8.4		0.8–8.4	0.8–8.4	
VTQ ratio			0.728			0.008			0.028
Mean,	1.9 ± 1.4	1,8 ± 1.3		2.9 ± 2.4	1.8 ± 1.1		2.1 ± 1.6	1.6 ± 0.9	
Range (min–max)	0.5–7.2	0.7–7.5		1.2–7.5	0.5–7.2		0.5–7.5	0.7–5.1	
EI			0.881^#^			0.511^#^			0.937^#^
Score 1, *n* (%)	0 (0.0)	1 (1.5)		0 (0.0)	1 (0.9)		0 (0.0)	1 (1.7)	
Score 2, *n* (%)	9 (23.4)	14 (17.4)		2 (22.2)	21 (19.6)		12 (21.4)	11 (18.3)	
Score 3, *n* (%)	23 (51.1)	37 (52.1)		3 (33.3)	57 (53.3)		28 (50.0)	32 (53.3)	
Score 4, *n* (%)	10 (25.5)	22 (29.0)		4 (44.4)	28 (26.2)		16 (28.6)	16 (26.7)	

N, the number of nodules; ARFI, acoustic radiation force impulse; VTI, virtual touch tissue imaging; VAR, virtual touch tissue imaging area ratio; VTQ, virtual touch tissue quantification; EI, elasticity imaging; LNM, lymph node metastasis; ETE, extrathyroidal extension;

# *Fisher's Exact Test*.

### Multivariate Logistic Regression Analysis

As shown in Table 4 (Figures 2, 3), multivariate logistic regression analysis showed that rich internal flow (OR: 6.66, 95% CI: 1.97–22.53, *P* = 0.002) was the strongest independent predictor for central LNM, followed by male sex (OR: 4.22, 95% CI: 1.65–10.74, *P* = 0.003), halo sign absence (OR: 2.78, 95% CIs: 1.09–7.08, *P* = 0.032); whereas VTI grading, VAR, VTQ, VTQ ratio, and EI were not significantly associated with central LNM.

**Table 4:** Multivariate logistic regression analysis in predicting prognosis.

	**Odds ratios**	**95% CIs**	***P***
**Central LNM**			
Male sex^d^	4.215	1.654–10.742	0.003
Halo sign absence^a^	2.777	1.089–7.081	0.032
Rich internal flow^b^	6.662	1.970–22.530	0.002
**Lateral LNM**			
VTQ ratio	1.569	1.077–2.285	0.019
**ETE**			
Male sex^d^	3.285	1.269–8.504	0.014
Halo sign absence^a^	2.903	1.159–7.272	0.023
Rich internal flow^b^	6.329	2.000–20.027	0.002
VTQ ratio^c^	1.625	1.106–2.388	0.013

*LNM, lymph node metastasis; VAR, virtual touch tissue imaging area ratio; VTQ, virtual touch tissue quantification; ETE, extrathyroidal extension*.

a *Represents halo sign absence*.

b *Represents rich internal flow*.

c *Represents VTQ ratio*.

d *Represents male sex*.

The risk for ETE was independently associated with rich internal flow (OR: 6.33, 95% CI: 2.00–20.03, *P* = 0.002), male sex (OR: 3.29, 95% CI: 1.27–8.50, *P* = 0.014), halo sign absence (OR: 2.90, 95% CI: 1.16–7.27, *P* = 0.023), and VTQ ratio (OR: 1.63, 95% CI: 1.11–2.39, *P* = 0.013); whereas VTI grading, VAR, VTQ and EI were not significantly associated with ETE.

VTQ ratio (OR: 1.57, 95% CI: 1.08–2.29, *P* = 0.019) was identified to be the only independent predictor for the lateral LNM.

### ROC Analyses

The results were showed in Table 5. The overall diagnostic performance of independent predictors for central LNM, such as rich internal flow (Az: 0.64; 95% CI: 0.54–0.74; sensitivity: 42.5%, specificity: 74.3%) was superior to halo sign absence (Az: 0.56, 95% CI: 0.45–0.67; sensitivity: 69.0%, specificity: 43.2%). Male sex was another predictor (Az: 0.62, 95% CI: 0.51–0.73; sensitivity: 42.9%, specificity: 81.1%). The combination of significant US features had an Az of 0.59 (95% CI: 0.48–0.69), a sensitivity of 23.8%, and a specificity of 90.5% in the prediction of central LNM. By combining male sex with the significant US features, the specificity increased to 98.6%.

**Table 5:** ROC analyses of the independent variables in identifying prognosis from patients with papillary thyroid cancer.

	**Az**	**95%CIs**	**Cut-off value**	**Sensitivity (%)**	**Specificity (%)**
**Central LNM**					
Male sex^d^	0.620	0.510–0.729	Male	42.9	81.1
Halo sign absence^a^	0.561	0.453–0.669	Halo sign absence	69.0	43.2
Rich internal flow^b^	0.641	0.538–0.744	Rich internal flow	42.5	74.3
a+b	0.572	0.460–0.683	a+b	23.8	90.5
a+b+d	0.529	0.418–0.640	a+b+d	7.1	98.6
**Lateral LNM**					
VTQ ratio	0.689	0.526–0.852	1.14	100.0	36.4
**ETE**					
Male sex^d^	0.596	0.492–0.700	Male	37.5	81.7
Halo sign absence^a^	0.547	0.442–0.652	Halo sign absence	66.1	43.3
Rich internal flow^b^	0.634	0.533–0.735	Rich internal flow	42.9	76.7
VTQ ratio^c^	0.608	0.504–0.712	1.21	69.6	53.3
a+b	0.565	0.460–0.670	a + b	21.4	91.7
a+b+c	0.554	0.449–0.659	a+b+c	12.5	98.3
a+b+c+d	0.509	0.403–0.615	a+b+c+d	1.8	100.0

*LNM, lymph node metastasis; CIs, confidence intervals; VAR, virtual touch tissue imaging area ratio; VTQ, virtual touch tissue quantification; ETE, extrathyroidal extension*.

a *Represents halo sign absence*.

b *Represents rich internal flow*.

c *Represents VTQ ratio*.

d *Represents male sex*.

For ETE, the diagnostic performance of rich internal flow (Az: 0.63; 95% CI: 0.53–0.74; sensitivity: 42.9%, specificity: 76.7%) was superior to other predictors such as VTQ ratio (Az: 0.61, 95% CI: 0.50–0.71; sensitivity: 69.6%, specificity: 53.3%), male sex (Az: 0.60, 95% CI: 0.49–0.70; sensitivity: 37.5%, specificity: 81.7%), and halo sign absence (Az: 0.55, 95% CI: 0.44–0.65; sensitivity: 66.1%, specificity: 43.3%). The optimal cutoff value of VTQ ratio was at least 1.21. The combination of significant US features had Az of 0.57 (95% CI: 0.46–0.67), a sensitivity of 21.4%, a specificity of 91.7%. By combining VTQ ratio with the significant US features, the specificity increased to 98.3%. By combining male sex with the significant US features and VTQ ratio, the specificity increased to 100%.

VTQ ratio was the only independent predictor for lateral LNM, which had an Az of 0.69 (95% CI: 0.53–0.85), a sensitivity of 100.0%, and a specificity of 36.4% in the prediction of lateral LNM. The optimal cutoff value of VTQ ratio was at least 1.14.

## Discussion

Although BRAF^V600E^ mutation has been shown to be helpful for identifying aggressive clinic pathologic characteristics and poor prognostication of PTC, the sensitivities and specificities were low (4–8). ETE including cervical LNM and extra-thyroid infiltration in patients with PTC was common and has been regarded to be associated with local recurrence, and regional or distant metastasis due to survival decreased (10, 11). Cervical LNM and extra-thyroid infiltration were the important factors to indicate the extent of surgery such as hemi-thyroidectomy vs. total thyroidectomy and no dissection vs. cervical LN dissection, especially in the high-risk PTC group.

As clinical important supplementary technique, US features of thyroid nodules in evaluating the malignancy have already been determined (12, 20, 28, 29). However, the relation of preoperative US findings and prognosis was reported few (15–17). In the preoperative US, we found that rich internal flow (OR: 6.66, *P* = 0.002) was the strongest independent factors to predict central LNM and ETE, although the sensitivities (42.5–42.9%) and specificities (74.3–76.7%) were moderate. In many studies reported that internal flow, capsule contact, and capsule involvement on US were associated with cervical LNM and ETE (15–17, 30). In univariate analysis, predictive factor of capsule involvement was associated with ETE, however, was not independent predictor after adjusted by multivariate logistic analysis. In a study by Kim et al. (31), tumor size was associated with ETE or cervical LNM, but capsule involvement was not associated with cervical LNM. In the present study, tumor size was not significantly associated with poor prognosis, which was in agreement with the report that the incidence of cervical LNM was not significantly difference between tumors ≤10 mm and tumors larger than 10 mm (32). As another US feature, halo sign absence (OR: 2.78 and 2.90, *P* = 0.032 and 0.023) was also associated with central LNM or ETE. None of the studies had found it as a predictor for poor prognosis until present. Likewise, Kim and Park et al. (9, 33) reported that US suspicious features were significantly associated with recurrence or lateral LNM. However, the above studies did not include the independent variable of halo sign absence. Recently, the association between infiltrative margins found on histopathology and cervical LNM of PTC has been confirmed in several reports (34–36), which may be the basic mechanism for halo sign absence in predicting central LNM or ETE. In the present study, US features such as echogenicity, micro-calcification, poorly defined margin, taller than wide ship, irregular shape, capsule contact, and solid nodule were not associated with poor prognosis in the positive BRAF^V600E^ mutation group. These results show that some important US predictors might be under debate in certain circumstances. Thus, new supplementary techniques may be needed to optimize the prognostic evaluation.

In the present study, EI and VTI were not helpful in predicting prognosis, which are similar to the results in previous studies (18, 19, 37). The value of SWV was higher in the patients with lateral LNM than in that without lateral LNM (*P* < 0.05), but a hard tumor on VTQ was not a predictor for lateral LNM after adjusted by multivariate logistic analysis. In contrast, VTQ ratio (OR: 1.57, 1.63, *P* < 0.05) was found to be associated with not only lateral LNM but also ETE. The sensitivity of VTQ ratio was up to 100% in predicting lateral LNM. These findings may be explained by the adjacent thyroid parenchyma stiffness of the target tumor reflected from VTQ ratio, and VTI and VTQ only reveal the stiffness of tumor self. To our knowledge, VTQ ratio is a feasible parameter for ARFI imaging, which is also the first report as a predictor for lateral LNM or ETE in the high-risk PTC group.

In addition, the predictive performance (Az, 0.57) was not improved by combination of the predictors on US such as halo sign absence or rich internal flow compared with those alone in predicting central LNM (Az, 0.56–0.64). However, the specificity of the US features combination increased to 90.5% was superior to that of US features alone (43.2–74.3%). For ETE, the specificity of the combination with VTQ ratio (98.3%) was not only superior to that of US features alone (43.3–76.7%) but also superior to that of US features combination (91.7%). In our additional study, the diagnostic performance of all independent predictors in patients with positive BRAFV600E mutation was similar to that in patients with negative BRAFV600E mutation (all *P* > 0.05). These findings were important in clinical practice, which may avoid unnecessary cervical LN dissection in the high-risk PTC group, and aid to select low-risk thyroid cancer patients for active surveillance or surgery.

In our series, male sex seemed to be other predictors of high risk central LNM or ETE based on the multivariate logistic analysis, as found in previous study (38). Combining US features with male sex, the specificity up to 98.6% was better to US features alone or combination (43.2–90.5%) for predicting the central LNM. Meanwhile, the specificities of VTQ ratio, US features and male sex combination increased to 100.0% were also better to those of VTQ ratio or US features alone or that of VTQ ratio and US features combination (43.3–98.3%) for predicting ETE. The finding may provide the reference for clinical practice.

We acknowledge several limitations of this study. First, our study was only enrolled the patients with positive BRAF^V600E^ mutation, so selection bias may be present. Second, surrogate endpoints for prognosis was used in our study because of low mortality of patients with PTC. Third, lateral LN dissection was performed based on clinical and radiological suspicious cervical LNM. The patients who hadn't performed lateral compartment dissection were regarded as negative, and at least 1-year follow-up was available. Finally, the source of the cervical LNM or ETE was undetermined in patients with multiple and bilateral foci. The patients with multiple cancers weren't excluded to mimic the real clinical situation.

## Conclusion

VTQ ratio on ARFI imaging, rich internal flow and halo sign absence on US are the predicting prognostic factors in the PTC patients with BRAF^V600E^ Mutation. The specificities were significantly increased by combining ARFI imaging and US features, which has a potential to avoid unnecessary therapeutic neck dissection in the high-risk PTC patients.

## Data Availability Statement

In our study, direct sequencing was performed for BRAF mutation analysis from FNAB sample with patient's nodule, which was not performed by animal's nodule. Therefore, ethics committee confirmed that the sequencing data cannot be made publicly available for ethical or privacy restrictions.

## Ethics Statement

This study was carried out in accordance with approved guidelines. The ethics committee of university hospital approved this prospective study and informed consent was obtained from all patients for all experimental protocol before their examination. All subjects gave written informed consent in accordance with the Declaration of Helsinki. The protocol was reviewed andapproved by the Ethic Committee of Tenth People's Hospital-Research Institute, Shanghai, China.

## Author Contributions

J-MX was responsible for study design, data collection, data analysis, and writing of the manuscript. Y-JC contributed to study design and writing of the manuscript. Y-JC and Y-YD participated in data acquisition and compiling. MC contributed to study design and edited the manuscript. All authors approved the final version of the manuscript.

### Conflict of Interest

The authors declare that the research was conducted in the absence of any commercial or financial relationships that could be construed as a potential conflict of interest.

## References

[1] Jemal A Bray F Center MM Ferlay J Ward E Forman D. Global cancer statistics. CA Cancer J Clin. (2011) 61:69–90. 10.3322/caac.20107 21296855

[2] Mazzaferri EL Kloos RT. Clinical review 128: current approaches to primary therapy for papillary and follicular thyroid cancer. J Clin Endocrinol Metab. (2001) 86:1447–63. 10.1210/jcem.86.4.7407 11297567

[3] Hughes DT Haymart MR Miller BS Gauger PG Doherty GM. The most commonly occurring papillary thyroid cancer in the United States is now a microcarcinoma in a patient older than 45 years. Thyroid. (2011) 21:231–6. 10.1089/thy.2010.0137 21268762

[4] Xing M Alzahrani AS Carson KA Shong YK Kim TY Viola D. Association between BRAF V600E mutation and recurrence of papillary thyroid cancer. J Clin Oncol. (2015) 33:42–50. 10.1200/JCO.2014.56.8253 25332244 PMC4268252

[5] Alzahrani AS Xing M. Impact of Iymph node metastases identified on central neck dissection (CND) on the recurrence of papillary thyroid cancer: potential role of *BRAF*^V600E^ mutation in defining CND. Endocr Relat Cancer. (2013) 20:13–22. 10.1530/ERC-12-0309 23132792 PMC3779438

[6] Elisei R Viola D Torregrossa L Gianniui R Romei C Ugolini E. The BRAF (V600E) mutation is an independent, poor prognostic factor for the outcome of patients with low-risk intrathyroid papillary thyroid carcinoma: single-institution results from a large cohort study. J Clin Endocrinol Metab. (2012) 97:4390–8. 10.1210/jc.2012-1775 23066120

[7] Li C Lee KC Schneider EB Zeiger MA. BRAF V600E mutation and its association with clinicopathological features of papillary thyroid cancer: a meta-analysis. J Clin Endocrinol Metab. (2012) 97:4559–70. 10.1210/jc.2012-2104 23055546 PMC3513529

[8] Sun J Zhang J Lu JL Gao J Ren X Teng L. BRAF V600E and TERT promoter mutations in papillary thyroid carcinoma in chinese patients. PLoS ONE. (2016) 11:e0153319. 10.1371/journal.pone.0153319 27064992 PMC4827831

[9] Kim SY Kwak JY Kim EK Yoon JH Moon HJ. Association of preoperative US features and recurrence in patients with classic papillary thyroid carcinoma. Radiology. (2015) 277:574–83. 10.1148/radiol.2015142470 25955580

[10] Baek SK Jung KY Kang SM Kwon SY Woo JS Cho SH. Clinical risk factors associated with cervical lymph node recurrence in papillary thyroid carcinoma. Thyroid. (2010) 20:147–52. 10.1089/thy.2008.0243 19785522

[11] Lundgren CI Hall P Dickman PW Zedenius J. Clinically significant prognostic factors for differentiated thyroid carcinoma: a population-based, nested case-control study. Cancer. (2006) 106:524–31. 10.1002/cncr.21653 16369995

[12] Haugen BR Alexander EK Bible KC Doherty GM Mandel SJ Nikiforov YE. 2015 american thyroid association management guidelines for adult patients with thyroid nodules and differentiated thyroid cancer: the american thyroid association guidelines task force on thyroid nodules and differentiated thyroid cancer. Thyroid. (2016) 26:1–133. 10.1089/thy.2015.0020 26462967 PMC4739132

[13] Haberal I Celik H Gocmen H Akmansu H Yoruk M Ozeri C Which is important in the evaluation of metastatic lymph nodes in head and neck cancer: palpation, ultrasonography, or computed tomography? Otolarygol Head Neck Surg. (2004) 130:197–201. 10.1016/j.otohns.2003.08.025 14990916

[14] Ji YB Lee DW Song CM Kim KR Park CW Tae K. Accuracy of intraoperative determination of central node metastasis by the surgeon in papillary thyroid carcinoma. Otolarygol Head Neck Surg. (2014) 150:542–7. 10.1177/0194599813519405 24429357

[15] Xu JM Xu HX Li XL Bo XW Xu XH Zhang YF. A risk model for predicting central lymph node metastasis of papillary thyroid microcarcinoma including conventional ultrasound and acoustic radiation force impulse elastography. Medicine. (2016) 95:e2558. 10.1097/MD.0000000000002558 26817907 PMC4998281

[16] Xu JM Xu XH Xu HX Zhang YF Guo LH Liu LN. Prediction of cervical lymph node metastasis in patients with papillary thyroid cancer using combined conventional ultrasound, strain elastography, and acoustic radiation force impulse (ARFI) elastography. Eur Radiol. (2016) 26:2611–22. 10.1007/s00330-015-4088-2 26560715

[17] Nam SY Shin JH Han BK Ko EY Ko ES Hahn SY Preoperative ultrasonographic features of papillary thyroid carcinoma predicts biological behavior. J Clin Endocrinol Metab. (2013) 98:1476–82. 10.1210/jc.2012-4072 23463652

[18] Moon HJ Kim EK Yoon JH Kwak JY. Clinical implication of elastography as a prognostic factor of papillary thyroid microcarcinoma. Ann Surg Oncol. (2012) 19:2279–87. 10.1245/s10434-011-2212-3 22246427

[19] Park YJ Kim JA Son EJ Youk JH Park CS. Quantitative shear wave elastography as a prognostic implication of papillary thyroid carcinoma (PTC): elasticity index can predict extrathyroidal extension (ETE). Ann Surg Oncol. (2013) 20:2765–71. 10.1245/s10434-013-2927-4 23463092

[20] Xu JM Xu XH Xu HX Zhang YF Zhang J Guo LH. Conventional US, US elasticity imaging, and acoustic radiation force impulse imaging for prediction of malignancy in thyroid nodules. Radiology. (2014) 272:577–86. 10.1148/radiol.14132438 24689885

[21] Zhao CK Xu HX Xu JM Sun CY Chen W Liu BJ. Risk stratification of thyroid nodules with Bethesda category III results on fine-needle aspiration cytology: the additional value of acoustic radiation force impulse elastography. Oncotarget. (2017) 8:1580–92. 10.18632/oncotarget.13685 27906671 PMC5352079

[22] Xu JM Xu HX Zhang YF Guo LH Liu LN Bo XW. Virtual touch tissue imaging for differential diagnosis of thyroid nodules: the additional value of area ratio. J Ultrasound Med. (2016) 35:917–926. 10.7863/ultra.15.06002 27022168

[23] Bhatia KS Tong CS Cho CC Yuen EH Lee YY Ahuja AT. Shear wave elastography of thyroid nodules in routine clinical practice: preliminary observations and utility for detecting malignancy. Eur Radiol. (2012) 22:2397–406. 10.1007/s00330-012-2495-1 22645042

[24] Lim DJ Luo S Kim MH Ko SH Kim Y. Interobserver agreement and intraobserver reproducibility in thyroid ultrasound elastography. AJR Am J Roentenol. (2012) 198:896–901. 10.2214/AJR.11.7009 22451558

[25] Asteria C Giovanardi A Pizzocaro A Cozzaglio L Morabito A Somalvico F. US-elastography in the differential diagnosis of benign and malignant thyroid nodules. Thyroid. (2008) 18:523–31. 10.1089/thy.2007.0323 18466077

[26] Rubaltelli L Corradin S Dorigo A Stabilito M Tregnaghi A Borsato S. Differential diagnosis of benign and malignant thyroid nodules at elastosonography. Ultraschall Med. (2009) 30:175–9. 10.1055/s-2008-1027442 18496776

[27] Tuttle RM Morris LF Haugen B Shah JP Sosa JA Rohren EM Thyroid-differentiated and anaplastic carcinoma (Chapter 73). In: Amin MB Edge SB Greene F AJCC Cancer Staging Manual, 8th Edn. New York, NY: Springer International Publishing (2017). p. 425–434.

[28] Moon HJ Sung JM Kim EK Yoon JH Youk JH Kwak JY. Diagnostic performance of gray-scale US and elastography in solid thyroid nodules. Radiology. (2012) 262:1002–13. 10.1148/radiol.11110839 22357900

[29] Moon WJ Jung SL Lee JH Na DG Baek JH Lee YH. Benign and malignant thyroid nodules: US differentiation-multicenter retrospective study. Radiology. (2008) 247:762–70. 10.1148/radiol.2473070944 18403624

[30] Jacquot-Laperrière S Timoshenko AP Dumollard JM Peoch M Estour B Martin C. Papillary thyroid microcarcinoma: incidence and prognostic factors. Eur Arch Otorhinolaryngol. (2007) 264:935–9. 10.1007/s00405-007-0290-4 17431661

[31] Kim SS Lee BJ Lee JC Kim SJ Lee SH Jeon YK. Preoperative ultrasonographic tumor characteristics as a predictive factor of tumor stage in papillary thyroid carcinoma. Head Neck. (2011) 33:1719–26. 10.1002/hed.21658 22076977

[32] Arora N Turbendian HK Kato MA Moo TA Zarnegar R Fahey TJ. Papillary thyroid carcinoma and microcarcinoma: is there a need to distinguish the two? Thyroid. (2009) 19:473–7. 10.1089/thy.2008.0185 19348582

[33] Park VY Kim EK Moon HJ Yoon JH Kwak JY The thyroid imaging reporting and data system on US, but not the BRAFV600E mutation in fine-needle aspirates, is associated with lateral lymph node metastasis in PTC. Medicine. (2016) 95:e4292 10.1097/MD.0000000000004292 27442672 PMC5265789

[34] Jung YY Lee CH Park SY Park HJ Min HS Won JK. Characteristic tumor growth patterns as novel histomorphologic predictors for lymph node metastasis in papillary thyroid carcinoma. Hum Pathol. (2013) 44:2620–7. 10.1016/j.humpath.2013.07.025 24139209

[35] Kim KJ Hong SW Lee YS Kim BW Lee SC Chang HS. Tumor margin histology predicts tumor aggressiveness in papillary thyroid carcinoma: a study of 514 consecutive patients. J Korean Med Sci. (2011) 26:346–51. 10.3346/jkms.2011.26.3.346 21394301 PMC3051080

[36] Jung CK Kang YG Bae JS Lim DJ Choi YJ Lee KY. Unique patterns of tumor growth related with the risk of lymph node metastasis in papillary thyroid carcinoma. Mod Pathol. (2010) 23:1201–8. 10.1038/modpathol.2010.116 20543822

[37] Jin ZQ Lin MY Hu WH Li WY Bai SJ. Gray-scale Ultrasonography combined with elastography imaging for the evaluation of papillary thyroid microcarcinoma: as a prognostic clinicopathology factor. Ultrasound Med Biol. (2014) 40:1769–77. 10.1016/j.ultrasmedbio.2014.02.015 24768485

[38] Cho JK Kim JY Jeong CY Jung EJ Park ST Jeong SH. Clinical features and prognostic factors in papillary thyroid microcarcinoma depends on age. J Korean Surg Soc. (2012) 82:281–7. 10.4174/jkss.2012.82.5.281 22563534 PMC3341476

